# Supporting change in chronic disease risk behaviours for people with a mental illness: a qualitative study of the experiences of family carers

**DOI:** 10.1186/s12889-018-5314-z

**Published:** 2018-03-27

**Authors:** Jacqueline M. Bailey, Vibeke Hansen, Paula M. Wye, John H. Wiggers, Kate M. Bartlem, Jennifer A. Bowman

**Affiliations:** 1 School of Psychology, Faculty of Science and Information Technology, The University of Newcastle, University Drive, Callaghan, NSW 2308 Australia; 2 Hunter Medical Research Institute, Clinical Research Centre, Lot 1 Kookaburra Circuit, New Lambton Heights, NSW 2305 Australia; 3 University Centre for Rural Health, School of Public Health, University of Sydney, Uralba Street, Lismore, NSW 2480 Australia; 4 Population Health, Hunter New England Local Health District, Booth Building, Wallsend Health Services, Longworth Avenue, Wallsend, NSW 2287 Australia; 5 School of Medicine and Public Health, Faculty of Health and Medicine, The University of Newcastle, University Drive, Callaghan, NSW 2308 Australia

**Keywords:** Chronic disease risk behaviours, Caregiver, Health behaviour, Mental illness, Smoking, Alcohol, Nutrition, Physical activity

## Abstract

**Background:**

People with a mental illness experience greater chronic disease morbidity and mortality, and associated reduced life expectancy, compared to those without such an illness. A higher prevalence of chronic disease risk behaviours (inadequate nutrition, inadequate physical activity, tobacco smoking, and harmful alcohol consumption) is experienced by this population. Family carers have the potential to support change in such behaviours among those they care for with a mental illness. This study aimed to explore family carers’: 1) experiences in addressing the chronic disease risk behaviours of their family members; 2) existing barriers to addressing such behaviours; and 3) perceptions of potential strategies to assist them to provide risk behaviour change support.

**Methods:**

A qualitative study of four focus groups (*n* = 31), using a semi-structured interview schedule, was conducted with carers of people with a mental illness in New South Wales, Australia from January 2015 to February 2016. An inductive thematic analysis was employed to explore the experience of carers in addressing the chronic disease risk behaviours.

**Results:**

Two main themes were identified in family carers’ report of their experiences: firstly, that health behaviours were salient concerns for carers and that they were engaged in providing support, and secondly that they perceived a bidirectional relationship between health behaviours and mental well-being. Key barriers to addressing behaviours were: a need to attend to carers’ own well-being; defensiveness on behalf of the family member; and not residing with their family member; with other behaviour-specific barriers also identified. Discussion around strategies which would assist carers in providing support for health risk behaviours identified a need for improved communication and collaboration between carers and health services accessed by their family members.

**Conclusions:**

Additional support from general and mental health services accessed by family members is desired to assist carers to address the barriers to providing behaviour change support. Carers have the potential to support and extend health service interventions aimed at improving the chronic disease risk behaviours of people with a mental illness but may require additional information, and collaboration from services. Further research is needed to explore these constructs in a large representative sample.

## Background

People with a mental illness experience increased rates of preventable morbidity, mortality and reduced life expectancy [1–5], primarily due to higher rates of chronic disease [1, 6]. This preventable burden of illness is associated with a greater prevalence of the primary behavioural risks for chronic disease: inadequate nutrition, inadequate physical activity, tobacco smoking, and harmful alcohol consumption [6–9]. Previous research has consistently reported a high engagement in all four chronic disease risk behaviours by people with a mental illness [10–17]. For example, the smoking prevalence of people with a mental illness is at least two to three times that of the general population [18, 19]; however the prevalence has been reported as high as 80–90% among people with psychotic and substance abuse disorders [20–23]. Despite high levels of engagement in risk behaviours, people with a mental illness have expressed interest in improving their risk behaviours and in receiving assistance from mental health services to improve such behaviours [17, 24–28]. The need to address these disparities and provide support to change risk behaviours has been acknowledged as a priority by national and international governments and peak mental health entities [29–33].

Many people with a mental illness receive regular support from unpaid informal carers [34–39]. For example, approximately 43.5 million people (18.2% of the population) in the United States and 5 million (12%) people in the UK are informal carers, with 21% (USA) and 13% (UK) of those caring for a person with a mental illness [36, 39]. Further, in Australia, 2.4 million people (15% of the population) are estimated to provide care to a relative with a mental illness [37]. Family carers can provide emotional, social, functional and financial support, including tasks such as: interacting with health care and other services; participating in decisions regarding medical care; and supporting and/or extending health care interventions in the home environment [29, 40, 41]. Clinical guidelines and policies recommend that family carers are included in all aspects of care provision for people with a mental illness [40, 42, 43]; with such recommendations aiming to provide a holistic approach to mental health care provision and to increase the effectiveness of treatments and interventions provided by health care services [29, 44, 45].

A review of the literature of studies exploring the role family carers may have in addressing the chronic disease risk behaviours of those they care for identified five studies. Three qualitative studies suggested that carers are actively engaged in this activity [41, 46, 47]. For example, a study of 13 carers in the United States found that the majority reported actively supporting weight loss through encouraging exercise, healthy food grocery shopping and meal preparation for their family member with a mental illness [41]. Similarly, a South African study of 8 family caregivers of people with a mental illness reported that most caregivers purchased, prepared and served food to their family member every day [47]. An Australian study of 12 carers of smokers with a mental illness reported that carers were actively trying to regulate and manage the consumption of tobacco by their family member [46]. Additionally, a descriptive correlational study of 27 family carers supporting an adult with schizophrenia and diabetes mellitus from the United States reported 89% of carers prepared meals for their family member with 78% preventing high-fat, high-sugar food intake; 22% encouraged their family member to stop drinking alcohol; and 59% assisted their family member with exercise [48]. Finally, a quantitative Australian study of 144 family carers of adults with a mental illness reported the majority of carers tried all or most of the time to positively influence: fruit and vegetable consumption (63.8%), physical activity (60.3%), smoking (56.3%), and alcohol consumption (56.2%) [49].

Three of the identified studies were also the only previous research that reported potential barriers to carers addressing the chronic disease risk behaviours of the person they care for, and possible strategies for overcoming them [41, 46, 49]. The qualitative study conducted in Australia found a dissonance between carer concerns for the negative health effects of smoking and the autonomy of their family member, with some carers reporting facilitating access to cigarettes rather than supporting cessation attempts [46]. Additionally, the study identified a lack of communication between mental health services and carers as a barrier to the provision of care regarding smoking, with carers reporting a need for such services to communicate and collaborate with them on the provision of smoking cessation strategies [46]. Similarly, in the qualitative study conducted in the United States, carers indicated a need for guidance from health care professionals regarding strategies to promote weight loss by their family member [41]. The quantitative Australian study assessed carers’ perceptions of their role and ability in addressing the four health risk behaviours which could indicate potential barriers to addressing risk behaviours [49]. The majority of carers felt confident to talk to the person they cared for about each of the four health behaviours (51.7%–76.6%) and thought they had the knowledge and skills to encourage healthy behaviours (62.5%–83.3%) however, fewer felt it was possible to have a positive influence on each behaviour (29.5%–51.1%) and a considerable proportion reported encouraging healthy behaviours for the person they cared for could harm their relationship (32.2%–58.2%) [49]. These studies did not identify a broad range of barriers to carer provision of support for modifying such behaviours, nor did they identify supportive strategies to increase carer capacity to support behaviour change.

Given these gaps in evidence, an exploratory qualitative study was conducted to explore family carers’:

- Experiences in addressing multiple chronic disease risk behaviours of their family members (inadequate nutrition, inadequate physical activity, tobacco smoking, and harmful alcohol consumption);
- Existing barriers to addressing such risk behaviours;
- Perceptions of potential strategies to assist them to provide risk behaviour change support.

## Methods

### Design and setting

An exploratory focus group study of family carers of people with a mental illness was undertaken employing purposive and convenience sampling techniques within one non-metropolitan region in New South Wales, Australia. While focus groups can be susceptible to social desirability bias, focus group methodology is widely used to explore participants’ experiences of illness and health services [50]. Further, advantages of the focus group methodology include: encouragement of participation by individuals who are disinclined to participate in an individual interview; and the generation of a rich discussion and exploration of the phenomenon through both shared and divergent experiences among group participants [50, 51]. The study was approved by the Hunter New England Human Research Ethics Committee (No. 14/10/15/4.04) and was registered with the University of Newcastle’s Human Research Ethics Committee (No. H-2015-0387).

### Participants and recruitment

Family carers were recruited through established mental health carer support groups provided by either community mental health services or a local carer support organisation [52]. The facilitators of the support groups were approached by telephone and email and provided a brochure and information statement for support group attendees. The facilitators organised for a focus group to be conducted during a scheduled support group meeting with members who agreed to participate. Support group members were eligible to participate in the focus group if they were: 18 years or older and identified themselves as a carer for someone with any mental illness over 18 years; and were not employed to support that individual.

### Procedure

Four semi-structured focus groups of approximately 1 h duration were conducted, with each consisting of between five and eleven participants. Participants were reimbursed for their time, travel and parking expenses incurred through participation in the study to the value of a $15 grocery voucher. Author JAB facilitated all focus groups and author JMB (both female) observed and acted as a note taker. The facilitator, author JAB, has expertise in both quantitative and qualitative research methodologies. JAB’s research team have been exploring the broad area of addressing chronic disease risk behaviours among people with a mental illness for over a decade. The research reported in this paper was exploratory and the research team have no vested interest or bias in establishing anything about carers or the role they might have in this area. Carer support group facilitators were present in three of the four focus groups, but did not engage in the group discussion. The researchers had no prior involvement with the participants. Before the conduct of each focus group, JAB confirmed all participants had received, read and understood the information statement and answered any questions. JMB distributed consent forms to all participants and an information statement was provided to any participant that did not receive one prior to the group. Written informed consent was obtained from all participants prior to commencing the study. Participants were then asked to complete a short questionnaire prior to the focus group, taking on average 10 min to complete. A digital voice recorder was used with participants’ consent to record the focus group discussions. Authors JAB and JMB agreed that data saturation [53] was achieved after the conduct of four focus groups as no new content was produced in in the fourth group. This was additionally confirmed during the iterative code book development phase, where no new codes were generated from the last transcript, but rather led to code refinement.

### Measures

#### Participant characteristics

The questionnaire collected the following information regarding participant socio-demographic characteristics: age; gender; employment status; marital status; highest level of education achieved; postcode; and Aboriginal and/or Torres Strait Islander status. In addition, participants also reported both the number of years, and current hours per week usually engaged in their caring role; as well as the nature of the familial relationship with the person they cared for and whether they currently resided with them.

### Focus group

At the start of each focus group, the researchers identified the four risk behaviours that were of particular interest and invited carers to express their views and experiences relating to any or all of them. The four behaviours were not addressed systematically throughout the focus group proceedings but carers were prompted if particular behaviours were not arising in the discussion.

The semi-structured interview schedule contained the following questions to address the study aims:

1. What have been the issues for you in helping the person you care for manage their health behaviours?
2. What have you tried? What has been helpful at all?
3. What would work to support you in helping the person you care for manage their health behaviours?

### Analysis

The focus group recordings were transcribed verbatim and NVivo 11 [54] was used to assist with the organisational aspects of the analysis. Analysis was conducted by authors VH and JMB using an inductive thematic analysis approach as described by Braun and Clarke [55], with the additional use of a data-driven coding template. Authors VH and JMB independently generated initial codes from the transcript of the first focus group, engaged in one detailed discussion of discrepancies and reached consensus on a draft coding hierarchy with minimal effort. VH and JMB independently coded a second transcript using the draft coding hierarchy. A high level of consensus was reached between the two coders; where one meeting which addressed a small number of differences in coding was adequate to resolve any inconsistencies. Further discussion among all authors took place and formed the basis for the development of the final coding hierarchy. VH coded all transcripts using the coding hierarchy. Once the complete dataset was coded, themes were formed and a thematic structure was identified which was further assessed and modified to fit the complete dataset. VH and JMB developed detailed coding narratives including noting commonalities and grouping by risk behaviours.

## Results

A total of 31 of 32 invited carers (26 female and 5 male) consented to participate, with four focus groups being conducted. The participants were aged between 48 and 85 years (mean = 66.1 years). The focus groups consisted of 5, 7, 8, and 11 participants. The majority of carers were the parent of the family member they cared for (87.1%); and had been caring for their family member for more than 10 years (70.9%), with 41.9% in a caring role for more than 20 years. More than one third (38.7%) of the carers were currently residing with their family member, and a further 12.9% ‘sometimes’ did so (Table 1).

**Table 1:** Demographic and caring characteristics of participants

Characteristic	N	%
Age (mean(SD) range)	66.1 (10.1)	48–85
Gender		
Female	26	83.9
Employment status		
Employed full or part time	5	16.6
Performing unpaid work	6	20
Not currently employed- not seeking employment	19	63.4
Highest education level		
Completed Higher School Certificate or less	15	48.3
Certificate/diploma/university degree or higher	16	51.7
Marital status		
Married or living together in a relationship	18	58.1
Divorced/separated	8	25.8
Widowed	5	16.1
Aboriginal and/or Torres Strait Islander origin		
No	30	96.8
Unsure	1	3.2
Relationship to person with a mental illness		
Parent	27	87.1
Partner	2	6.5
Child	1	3.2
Sibling	1	3.2
Years in caring role		
Less than 1 year	1	3.2
1–2 years	0	0
3–10 years	8	25.8
11–20 years	9	29.1
More than 20 years	13	41.9
Hours per week in caring role		
Less than 10 h	11	35.4
11–37 h	12	38.8
38 h or more	8	25.8
Residing with person with a mental illness		
Yes	12	38.7
No	15	48.4
Sometimes	4	12.9

Throughout all focus groups, carers commented on the challenging nature of the caring role in general; noting that it entailed the provision of significant time, financial, practical and emotional support, as well as ensuring adherence to health care appointments and programs. Carers found it difficult to discuss their role in relation to chronic disease risk behaviours separately to their broader caring role: it was one aspect of care among many that they often felt themselves to carry sole responsibility for. Throughout the focus groups, carers often grouped together their discussion of risk behaviours, nutrition and physical activity, and smoking and alcohol, suggesting similarities in their addressing of such behaviours.

### Experiences in addressing the chronic disease risk behaviours

During exploration of family carers’ experiences, two main themes were identified: the first being that providing support for health risk behaviours was an important (salient) concern for carers and something they were engaged in doing; and the second being that carers perceived a bi-directional relationship between the health risk behaviours and the mental health of the person they cared for (Table 2). The perceived salience which carers placed on the risk behaviours was apparent and underpinned their efforts in attempting to address them. The salience of the risk behaviours as a problem to be addressed was closely tied to their perceived impact on the mental health and well-being of their family member.

**Table 2:** Themes and sub-themes from the data analysis

Theme and sub-themes
Experiences in addressing the chronic disease risk behaviours
- Salience of risk behaviours and interaction of risk behaviours with mental illness
- Nutrition and physical activity
- Smoking and alcohol
Existing barriers to addressing chronic disease risk behaviours - Nutrition and physical activity - Smoking and alcohol
Potential supportive strategies to address carers’ needs
- Current sources of support
- Interface between carers and health services

### The salience of the risk behaviours and their interaction with mental illness

#### Nutrition and physical activity

There was general consensus that both regular physical activity and adequate nutrition were salient components in achieving and maintaining not only physical (i.e., preventing or alleviating diabetes, sleep apnoea etc.), but also mental well-being; with diet the behaviour carers tried to address most often. Many carers reported the importance of regular physical activity as a component in stress management not only for their family members, but also for themselves; and as an element of cohesion or structure in the life of their family member. Furthermore, one carer stated that the maintenance of physical activity and adequate nutrition could aid mental illness symptom management. However, a few carers voiced a need to place a secondary importance on these risk behaviours at times, relative to managing acute mental health problems.

Nutrition and physical activity were seen to have a close interaction with mental illness, both in terms of the physiological or organic basis for the condition as well as its behavioural manifestations and symptomatology; where mental illness was seen to exert a strong influence on nutrition and physical activity. On one hand, there was a widespread understanding among carers that many psychotropic medications resulted in ‘inevitable’ weight gain (direct side effect); while others expressed an understanding that this was due to an effect via appetite regulation and/or cravings for carbohydrate rich foods (indirect side effect). One carer in particular had found the latter knowledge helpful, both for herself and her family member, as it facilitated a greater internal locus of control;

*“And it’s been really good for him to realise that it’s just not inevitable that if he’s on this particular medication that he will put on weight, that he actually has a choice about it.”* (Participant 13, female)

Nutrition, physical activity and weight problems/fluctuations were seen as closely tied to the state of the person’s mental health condition and how well it was managed. For physical activity, the adoption of, or motivation to engage in a physical activity routine was perceived to be influenced by the state of the person’s mental illness and/or psychotropic medication effectiveness. Similarly, many carers saw the challenges of adhering to adequate nutrition or maintaining a healthy weight as significantly compounded by illness characteristics such as a lack of insight;

*“I've got a problem with my son, that he will not eat anything that he thinks is going to put any weight on. That's a big worry to us. He went down to 37 kilos at one time, which was pretty drastic. That was many years ago. Because of his anxiety and paranoia, he is - he sees himself as being really overweight. You could not get him to eat a cake or a lolly or anything like that.”* (Participant 27, female)

#### Smoking and alcohol

While engaging in healthy nutrition and physical activity behaviours were seen to aid stress and mental illness symptom management, similarly carers noted that smoking had a calming effect for their family member and increased mental clarity, and family members would consume tobacco at higher levels in times of stress. Conversely, carers acknowledged that the consumption of alcohol had negative consequences for mental health. Regardless of whether engagement in each of the behaviours had positive or negative consequences for mental health, carers acknowledged the need at times to prioritise mental illness management above a health behaviour routine.

In terms of alcohol consumption by the person being cared for, the consistent message conveyed was a shared understanding that while alcohol clearly had significant negative consequences for the health and mental stability of their family member, it also played a role in generating feelings of ‘normality’ for them. The desire to consume alcohol by the family member was described in terms of their being driven by a need to “feel” and to “feel normal”. This sense of normality was described both in terms of their family member’s feelings of normal social functioning and social inclusion and acceptance;

*“…people would buy him drinks or he’d buy them drinks or like you know, I can’t believe that in my community that people would be so silly, but he’d say “I just want to feel normal”.”* (Participant 9, female)

Among those caring for a smoker, most described smoking to have been instigated by stress or anxiety and continued as a means of stress management. All carers expressed the view that nicotine had calming effects for their family member, while others mentioned cognitive effects such as a greater ability to think clearly. Others noted that smoking served as a diversion or hobby to occupy their family member’s time. The interaction between smoking and mental illness was seen to be bidirectional in nature, with the majority of carers, regardless of whether they cared for a smoker, agreeing that smoking often resulted in a lessening of mental health symptoms. Some discussed a perceived link between the uptake of smoking and the onset of mental illness symptoms or relapse, and noted that craving and consumption of tobacco was greater during onset or acute phases of the illness;

*“I noticed with my son that he probably had the odd cigarette-not around the time that guys usually smoke, you know in their teens and stuff, but much later-but I noticed around his time of diagnosis it just seemed to increase and increase and increase. And um, I also noticed that, obviously, like … in his un-wellness, he tends to go that, you know, shwooo, really drag on the cigarette and stuff.”* (Participant 9, female)

A few carers were aware of the effect changes in smoking status or nicotine intake levels could have on psychotropic medications.

### Existing barriers to addressing chronic disease risk behaviours

Carers mentioned three barriers which hindered their provision of support with risk behaviours in general. Firstly, the need for carers to attend to their own mental and physical health needs in order to have the resources and resilience to assist their family members was frequently cited. Despite this, carers’ own needs were often stated to be overlooked out of perceived necessity and priority of caring for their family member. Carers experienced a lack of attention and inadequate support for their own well-being; both within the context of the caring relationship and from health services accessed by their family member. Secondly, defensiveness on behalf of their family member such as: direct obstinate behaviours and attitudes; and a denial or un-readiness for change, which was mainly perceived as being caused by the mental illness and subsequent lack of insight. This influenced the extent to which carers could facilitate and support behaviour change. In general, almost all carers felt that providing prompting and motivation was required to bring about and maintain changes to health risk behaviours. Thirdly, the effectiveness of such strategies was perceived to be largely dependent on whether they lived with their family member; with those residing with their family member having increased awareness of engagement in risk behaviours and capacity to provide behaviour change support.

#### Nutrition and physical activity

The key barriers to promoting nutrition and physical activity were medication induced cravings, cost, and motivation. In addition to the challenges of promoting healthy nutrition generally, many carers talked of the difficulties of managing nutrition in the context of managing mental illness, such as attending to and managing medication induced cravings, using strategies such as locking of cupboards and the refrigerator at night.

A few carers voiced concern that it was comparatively more expensive to eat a healthy diet as opposed to take away, instant or processed foods. In addition, carers mentioned the inability of a lot of family members to manage money appropriately and to prioritise food over cigarettes or coffee. Motivation of the family member was one of the main barriers to their family members engaging in physical activity, with some perceiving this to be attributable to psychotropic medications. There was a widespread acknowledgement that disruption to a regular routine such as changes to medications, hospitals stays etc. often would have a detrimental impact on the motivation to engage in any regular physical activity.

Some carers sought to influence physical activity levels through exercising together, but noted that the responsibility of organising and motivating such activity inherently fell on them. For those whose family members’ condition allowed sufficient insight to facilitate an understanding of the importance of good physical health underpinning their mental health, carers appeared to be more engaged in overt behaviour support such as giving reminders. When caring for people who were acutely unwell or chronically lacking insight, more covert behaviour modification strategies were adopted;

*“Because he's got no insight whatsoever that he's unwell, so, for him, he thinks McDonald's is healthy food and fruit and vegetables and stuff is bad, it's the junk food. You can't reason with him whatsoever. So except for the few vegies that I might be able to hide in a meal, he doesn’t eat any vegetables, doesn’t eat any fruit at all.”* (Participant 24, female)

The experience of supporting improvements in their family members’ nutrition was a multi-faceted challenge and one understood to require a more holistic approach than would be required for someone without a mental illness. There was also widespread acknowledgement that disruption to a regular routine such as changes to medications or hospital stays often would have a detrimental impact on any regular physical activity engagement. Carers were facing a range of physiological, mental, and financial barriers to supporting behaviour change, as well as those relating to the dynamics within their carer role.

#### Smoking and alcohol

Whilst carers identified medication induced cravings, cost, and motivation as the fundamental barriers to promoting nutrition and physical activity; the barrier most mentioned to hinder smoking cessation support was engaging in contradictory behaviours concerning the supply of tobacco by carers to their family members; and denial by the family member impeded carers’ ability to promote reduced alcohol consumption.

A dominant barrier to assisting their family member to quit smoking was carer complicity in enabling the supply of tobacco to their family member despite their concern about the negative health impact of smoking. Some carers reported assisting their family member to smoke by providing money to purchase cigarettes, while others reported supplying their family member with cigarettes during an inpatient stay in a mental health service. The provision of cigarettes was in some instances based on an underlying belief in the calming effects of smoking, and for others an acknowledgement of their lack of reasonable ‘authority’ as current smokers themselves. Other frequently mentioned barriers to assisting in smoking cessation were the inability of their family member to remain abstinent from smoking during times of stress, and their family members’ prioritisation of smoking over psychiatric medications, food and other necessities;

*“[My son has] actually been selling his medication off… I've been giving him his [ten] tablets that have to last 10 days. I found out … three days later they'll disappear. So we had it out last night and found out that he's been selling them, just to have a cigarette.”* (Participant 21, female)

Many participants reported that their family member had previously tried various forms of nicotine replacement therapy with varying degrees of cessation success. All carers agreed that nicotine replacement therapy was an inadequate aid in permanent cessation, and insufficient to maintain abstinence during a mental health relapse, or stressful life event. Strategies that carers reported implementing to support cessation attempts or reduce tobacco consumption included: banning smoking inside the home and introducing competing financial needs (such as being able to afford petrol for the car). One carer, who herself was a smoker, described supporting cessation through a joint quit attempt;

*“I was smoking, I’ve actually only gave it up a month ago… again. I wasn’t smoking a lot... about 15 a day probably, which was less than I used to smoke… and [family member] gave it up at the same time. We both gave it up. And he stopped as well, and so did I.”* (Participant 1, female)

Few barriers to reducing the consumption of alcohol by the person being cared for were mentioned. Refusal to acknowledge the need to decrease/abstain from alcohol consumption due to interactions with mental illness and/or psychotropic medications on behalf of their family members, as well as social opportunities centred around alcohol were cited;

*“He even knows now that he shouldn’t [drink] with the medication but it hasn’t, you know like he, he says “I didn’t hear that”. It doesn’t matter who says it to him, “I didn’t hear that”. So he’s really quite able to deflect what he doesn’t want to hear.”* (Participant 11, female)

On the one hand carers expressed feeling compelled to support their family members to attend social opportunities even if alcohol was available, as their family members were known to experience social exclusion and withdrawal; while on the other hand some acknowledged the need to minimise alcohol consumption due to the negative overall impact it was known to have on their physical and mental health.

Despite prompting, there was little discussion about the strategies which were seen to assist the carer to address alcohol related problems. However, some carers briefly talked of strategies they employed such as “metering” out money, thereby preventing their family member from having large sums of money available to spend on alcohol, so in essence, preventing opportunities for consumption.

### Potential supportive strategies to address carers’ needs

Two main themes were identified within this aim: current sources of support and the interface between carers and health services. When discussing how carers felt they could be better assisted in supporting behaviour change in their family members many carers reported their current sources of support or previous experiences with various health services where they generally received suboptimal support. Carers then discussed the interface between carers and health services, reporting a need for improved communication and collaboration from services to carers.

### Current sources of support

Carers expressed the view that little information and services were available to support them in helping their family members change their health risk behaviours. No carers reported accessing existing general community supports for specific risk behaviours including telephone coaching support, such as the Quitline [56] service for smoking. While general practitioners, dieticians, mental health family workers, and the internet were all reported as sources from which information was sometimes derived, carer groups provided by mental health services were considered the most valued source of information and support;

*“Everything is covered here. You just get so much knowledge… Sometimes it doesn't come to you until weeks later that you picked up something that you're coping better with; that you've learnt from somebody else's experience … You can leave the group and you feel more positive.”*(Participant 18, female)

Health, fitness or community services were mentioned as having a positive influence where many carers reported their family member had accessed such a service to support engagement in physical activity. However, the benefits of such activity were only maintained for the length of time that the service was accessed. No services appeared to have had a role in facilitating sustainable behaviour change. One carer also stressed the importance of social inclusion and shared experience as a facilitator to participating in physical activity whereby a family member was motivated to engage in an exercise program conducted with other people with depression;

*“She was going through the Uni, they did a research program where it was under 25’s with a depression- they were doing an exercise program … there was a whole heap of other cutters*^1^ *there that they all had the shorts on and you could see the scars. And so my daughter felt normal, um, and that was really, really good and she was getting very motivated.”* (Participant 7, female)

While few carers talked of resources which facilitated their provision of nutrition support for their family member, a few talked of some helpful information received from family, support workers and general practitioners. There was however consensus that basic nutrition information targeted at the general population was of little use. Rather, it was seen as important that such information was specific, practical, hands-on and considered the very different set of parameters and contextual difficulties within which they operated.

### Interface between carers and health services

There were mixed views of the role of health services in supporting carers and their family members in risk behaviour change. Some carers talked of positive changes in both behaviours and attitudes of their family members as a result of services accessed via their National Disability Insurance Scheme plans [57], or while being hospitalised. Periods of hospitalisation were often referred to as a positive time for their family member, where positive routines and improved risk behaviours were established which, for some, extended to a beneficial sustained change post-discharge. Yet others shared their experiences of negative changes as a direct result of contact with health services, such as excessive unhealthy weight loss and taking up smoking;

*“She started smoking last year, just socially, but the last, when she was in hospital just before Christmas, um…yeah, and that’s when she started getting heavier and um… oh just before she went to hospital but coming out like on leave, she would look for cigarettes and everything and um, and then since coming out of hospital she does it to help relieve the anxiety, um, and she doesn’t cut now um, because she is sort of coping better with that.”* (Participant 7, female)

In almost all instances, these negative impacts of health services were perceived to be due to either a lack of service provider understanding of mental health issues or a lack of communication between carers and service providers. One of the more dominant themes referred to in relation to the role of health services in supporting risk behaviour change was an acknowledgment that the carer was sidelined (perceived to be mainly due to privacy laws) and not regarded as an important member of the “treatment team”. This lack of clear communication with the carer was seen to significantly hinder their ability to provide the continuity needed to facilitate sustainable behaviour changes;

*“I need to know what it is they’re saying to him so that I can actually support that… They need to communicate to the carer what it is so that we’re all on the one path.”* (Participant 13, female)

In addition, carers felt that their expertise, knowledge and instrumental role as carers and ‘health managers’ generally was not acknowledged, particularly by mental health services. Many carers recounted negative past experiences with services where they were not involved in treatment planning or diagnosis and their voice was perceived to not be heard by clinicians. A few also mentioned problems arising from compromised communication and lack of a useful dialogue between different health care providers (e.g., general practitioners and psychiatrists), impacting on their ability to change the risk behaviours of their family member;

*“I mean even our GP, he’s really good… but like even when [my son] had given up smoking and… said to him that he had given up smoking… he just said, “Oh I can’t you know, I can’t mess around with your medication.”… I mean I was the one who rang the psychiatrist and made the appointment and told him what had happened and like, he immediately said, “Oh right, yes, I agree with you, we need to get his bloods done straight away.” At the end of the day, all of us, we are the, we’re the Doctors, we’re the clinicians, we’re the dieticians, we really, really, really know what’s going on.”* (Participant 1, female)

Additionally, a few carers voiced complaints about allied health professionals either not sufficiently acknowledging the impact of mental health issues in dealing with risk behaviours, or not considering the diversity of mental health issues and the varying impact they can have on the efficacy and viability of potential strategies;

*“They seem to put mental illness and this is what you're like. This is what everybody is like. But they’re all so different… I think, trying to find people who can really understand that everybody's different in mental health. They just want to fit you into a box.”* (Participant 24, female)

Some also expressed frustration that the health system was perceived to not provide holistic care. That is, health care providers were seen to deal with the presenting problems while neglecting to address or acknowledge other related health issues, which were intricately linked to risk behaviours. Many carers desired health services to play a more active role and a more targeted role in close communication and cooperation with carers, such as provision of dietary support, and drug and alcohol rehabilitation.

## Discussion

This is the first exploratory study to investigate the four key chronic disease risk behaviours together and in depth from the perspectives of family carers; the barriers which influence carers capacity to address such risk behaviours; and potential supportive strategies which may assist carers to provide risk behaviour change support to their family member with a mental illness. Carers placed high importance on the chronic disease risk behaviours and were motivated to address them; with diet perceived as the behaviour they tried to address most often. All carers acknowledged an interaction between risk behaviours and mental illness symptoms where adhering to positive behavioural routines was complicated by mental illness characteristics and status. Despite implementing various strategies to encourage improvement of risk behaviours, carers acknowledged multiple barriers to doing so, with some differences in those mentioned for nutrition and physical activity, as compared to those for smoking and alcohol consumption. Medication induced cravings, cost and lack of motivation of the person being cared for were key barriers in promoting nutrition and physical activity, while the supply of tobacco by carers was a major barrier to promoting smoking cessation, and refusal to decrease/abstain from alcohol by the family member impeded carers ability to promote reduced alcohol consumption. Carers reported insufficient information and support currently available to assist them in supporting risk behaviour change and expressed a need for improved communication and collaboration between carers and health services to help them in doing so. Throughout the conduct of all focus groups it was at times difficult for carers to separate their discussion of experiences of their role in relation to chronic disease risk behaviours from their role in caring for the broader physical and mental health and other needs of their family member.

The understanding that the chronic disease risk behaviours are experienced differently by people with a mental illness, and hence require different intervention approaches has been reported previously in qualitative research of carers. Carers have previously reported the interconnectedness between physical and mental health where psychotropic medications can result in food cravings, weight gain and a loss of motivation to engage in physical activity [58]; and the perceived calming effects of nicotine on mental illness symptoms or improved cognition [46, 59]. Quantitative research has also reported differences in the experience of risk behaviours among people with a mental illness. For example, people with a mental illness experience higher nicotine dependence, smoke more cigarettes per day and are less likely to quit smoking compared to people without a mental illness [60–62]. Despite this, people with a mental illness have comparable levels to the general population of interest in changing their smoking behaviours and receiving support from mental health professional to do so [17, 24–28]. A limited and contrasting body of research exists on the effectiveness of population level approaches to address smoking behaviours among this population group with some research suggesting interventions are less effective but can result in some positive behaviour change [60]. However, two studies examining the impact of smoking cessation mass media campaigns on people with a mental illness determined such campaigns had no impact on smoking behaviours or intentions to quit [63, 64]. The literature is lacking in other aspects of experience such as the degree to which people with a mental illness lack access to services to support behaviour change and any barriers associated with access to services. Such findings suggest that mental health specific intervention strategies are required when addressing the chronic disease risk behaviours of clients with a mental illness and in supporting carers to play a role.

The prioritisation of mental health over physical health has previously been reported by carers [46, 58, 59] and mental health professionals [65, 66]. Carers have reported the need to stabilise mental health conditions with psychotropic medications, with the intention of addressing any negative consequences on nutrition and physical activity at a future point in time [58]. Similarly, the perception that nicotine can have a positive impact on mental illness symptoms, or that smoking cessation could result in mental illness relapse has been reported by both carers [46, 59] and mental health professionals [65, 66]. Such perceptions may suggest that carers and mental health professionals could benefit from the dissemination of evidence of the benefits of smoking cessation for both mental health and physical health [60, 67] and from the development of strategies addressing the perceived effects of smoking on mental health. Further, the results reinforce the need for mental health services to facilitate and provide adequate smoking cessation support to all clients who smoke as per policies and guidelines [68–70]; support which has previously been found to be suboptimal [71–75] and which was also noted to be so by some carers in the present study.

A lack of attention to the carer’s own physical and mental health needs in health service settings and in the caring relationship itself suggests a need for services to address the needs of carers in addition to those of the client with a mental illness. Previous studies have reported similar findings despite carer reported need for the maintenance of their own physical and mental well-being in order to continue to support their family member [58, 76]. While current mental health service policies acknowledge the need to address and support the physical and mental well-being of carers of people with a mental illness, [29, 77] carers in the present study reported receiving inadequate support for their own well-being.

Carers required additional support from health services in order to support behaviour change interventions; and expressed a need for health services to provide more holistic care for people with a mental illness. Carers in this study, and previous research, have identified a need for services to provide additional support in the form of increased information and collaboration to assist them in supporting family members in risk behaviour change [41, 46, 78]; as well as additional information and behaviour change support being provided directly to their family members [46, 59, 78]. The finding that carers’ awareness of their family member’s risk behaviours and ability to support or encourage behaviour change was often contingent on whether or not they resided together, suggests that when collaborating with carers, mental health services should ensure behaviour change support and advice is appropriate for the carer’s particular circumstances and the dynamics of the caring relationship. An increased provision of behaviour change information and support tailored to the needs of mental health clients, and a greater inclusion of carers within health service planning and interventions may result in more effective risk behaviour change interventions for people with a mental illness. New opportunities to better connect carers and mental health care providers should be sought, such as may arise for instance through the National Disability Insurance Scheme (NDIS), an agency aiming to support people with a range of disabilities to have a positive impact on everyday life [79–81]. Furthermore, in the large regional centre where the focus groups were conducted, a variety of services were available to carers and people with a mental illness, such as: primary care; non-government organisations and specialist mental health services. The current study did not systematically assess participants’ prior use of or perceived accessibility to such services. Future research could explore if carers perceive a lack of opportunity to access such services as a barrier to receiving adequate support from services.

Findings should be viewed in light of a number of study characteristics, including that focus groups were conducted through established carer support groups where sometimes a regular group facilitator was present. Such a presence may have coloured some reflections on the role and support provided by mental health services. Further, as participants were members of carer support groups, the extent to which responses are representative of the broader carer population is not known; although the demographic characteristics of participants are largely consistent with the characteristics of carers in Australia [82]. In addition, the large majority of carers were parents of the person they cared for (87.1%), and the dynamics explored through the carer adult-child relationship dynamic may not be representative of other caring relationship dynamics. Finally, the focus groups were conducted in one large non-metropolitan centre well serviced by a range of general and mental health support services, hence, such carers’ experiences with services in the current study may not reflect the experience of carers in more rural areas.

## Conclusions

Family carers were found to be trying to address the chronic disease risk behaviours of their family members but identified multiple barriers to supporting behaviour change. Additional support from general and mental health services accessed by their family members is desired. Carers have the potential to support and extend service interventions aimed at improving the chronic disease risk behaviours of people with a mental illness in the home environment but may require additional support, information, and collaboration from health services. Further research is needed to explore these constructs in a large representative sample. Future research could investigate carers’ need for services and support to assist them in supporting behaviour change and specifically the types of programs or interventions they would find useful. Additionally, future interventions could attempt to address shared risk behaviours between carers and people with a mental illness concurrently.

## Footnotes

1 People who engage in non-suicidal self-injury- the deliberate injuring of oneself without suicidal intent- by lacerating or piercing the skin with a knife or sharp object.

## References

[1] Brown S (2010). Twenty-five year mortality of a community cohort with schizophrenia. Br J Psychiatry.

[2] Laursen TM (2013). Life expectancy and death by diseases of the circulatory system in patients with bipolar disorder or schizophrenia in the Nordic countries. PLoS One.

[3] Wahlbeck K (2011). Outcomes of Nordic mental health systems: life expectancy of patients with mental disorders. Br J Psychiatry.

[4] Chang CK (2011). Life expectancy at birth for people with serious mental illness and other major disorders from a secondary mental health care case register in London. PLoS One.

[5] Lawrence D, Hancock K, Kisely S (2013). The gap in life expectancy from preventable physical illness in psychiatric patients in Western Australia: retrospective analysis of population based registers. BMJ.

[6] Callaghan RC (2014). Patterns of tobacco-related mortality among individuals diagnosed with schizophrenia, bipolar disorder, or depression. J Psychiatr Res.

[7] Scott D, Happell B (2011). The high prevalence of poor physical health and unhealthy lifestyle behaviours in individuals with severe mental illness. Issues Ment Health Nurs..

[8] Stanley F, Laugharne J (2014). The impact of lifestyle factors on the physical health of people with a mental illness: a brief review. Int J Behav Med.

[9] Australian Institute of Health and Welfare, Risk factors contributing to chronic disease. Cat no (2012). PHE157.

[10] Cook JA (2015). Health risks and changes in self-efficacy following community health screening of adults with serious mental illnesses. PLoS One.

[11] Hahn LA (2014). Inadequate fruit and vegetable intake in people with psychosis. Aust N Z J Psychiatry.

[12] Kilbourne AM (2009). Excess heart-disease-related mortality in a national study of patients with mental disorders: identifying modifiable risk factors. Gen Hosp Psychiatry.

[13] Kilian R (2006). Health behavior in psychiatric in-patients compared with a German general population sample. Acta Psychiatr Scand.

[14] Morgan VA (2014). Psychosis prevalence and physical, metabolic and cognitive co-morbidity: data from the second Australian national survey of psychosis. Psychol Med.

[15] Prochaska JJ (2014). Multiple risk-behavior profiles of smokers with serious mental illness and motivation for change. Health Psychol.

[16] Ussher M (2011). Cardiovascular risk factors in patients with schizophrenia receiving continuous medical care. Community Ment Health J.

[17] Bartlem K (2015). Chronic disease health risk behaviours amongst people with a mental illness. Aust N Z J Psychiatry.

[18] Lawrence D, Mitrou F, Zubrick S (2009). Smoking and mental illness: results from population surveys in Australia and the United States. BMC Public Health.

[19] AK M (2010). Smoking characteristics of adults with selected lifetime mental illnesses: results from the 2007 National Health Interview Survey. Am J Public Health.

[20] Reichler H (2001). Smoking among in-patients with drug-related problems in an Australian psychiatric hospital. Drug Alcohol Rev.

[21] Lineberry TW (2009). Population-based prevalence of smoking in psychiatric inpatients: a focus on acute suicide risk and major diagnostic groups. Compr Psychiatry.

[22] PJ K (2012). Prevalence of smoking and other health risk factors in people attending residential substance abuse treatment. Drug Alcohol Rev.

[23] de Leon J, Diaz FJ (2005). A meta-analysis of worldwide studies demonstrates an association between schizophrenia and tobacco smoking behaviors. Schizophr Res.

[24] Stockings E (2013). Readiness to quit smoking and quit attempts among Australian mental health inpatients. Nicotine Tob Res.

[25] Ussher M (2007). Physical activity preferences and perceived barriers to activity among persons with severe mental illness in the United Kingdom. Psychiatr Serv.

[26] Filia SL (2011). Health behaviour risk factors for coronary heart disease (CHD) in smokers with a psychotic disorder: baseline results. Mental Health and Substance Use.

[27] Moeller-Saxone K (2008). Cigarette smoking and interest in quitting among consumers at a psychiatric disability rehabilitation and support Service in Victoria. Aust N Z J Public Health.

[28] Siru R, Hulse GK, Tait RJ (2009). Assessing motivation to quit smoking in people with mental illness: a review. Addiction.

[29] NSW Mental Health Commission, Living well: a strategic plan for mental health in NSW. 2014, NSW Mental Health Comm: Sydney.

[30] NSW Department of Health (2009). Physical health care of mental health consumers.

[31] National Preventive Health Taskforce (2008). Australia: the healthiest country by 2020 - a discussion paper.

[32] Bobes-Garcia J (2012). Delphi consensus on the physical health of patients with schizophrenia: evaluation of the recommendations of the spanish societies of psychiatry and biological psychiatry by a panel of experts. Actas Esp Psiquiatr.

[33] National Institute for Health and Care Excellence, Psychosis and schizophrenia in adults (2014). The NICE guideline on treatment and management, N.C.C.F.M. Health, editor.

[34] Collings S (2009). Who cares for people with schizophrenia: family carers' health, circumstances and adjustment.

[35] Sinha M (2013). Spotlight on Canadians: results from the general social survey. Portrait of caregivers, 2012.

[36] AARP Public Policy Institute (2015). N.A.f.C., executive summary: caregiving in the U.S.

[37] Pirkis J (2010). Who cares? A profile of people who care for relatives with a mental disorder. Aust N Z J Psychiatry.

[38] Australian Bureau of Statistics (2008). A profile of carers in Australia, A.B.o. Statistics.

[39] NHS Information Centre Social Care Team. Survey of Carers in households 2009/2010. Leeds: NHE Information Centre for Health and Social Care; 2010.

[40] Office of the Chief Psychiatrist, Communicating with carers and families (2007). Information sharing for better outcomes. Delivering a healthy WA, D.O. Health.

[41] Aschbrenner KA (2014). A mixed methods exploration of family involvement in medical care for older adults with serious mental illness. Int J Psychiatry Med.

[42] NICE, Schizophrenia: Core interventions in the treatment and management of schizophrenia in primary and secondary care (updated edition) clinical guideline 82. Leicester: The British Psychological Society & The Royal College of Psychiatrists; 2010. 20704054

[43] National Mental Health Commission (2014). The national review of mental health programmes and services.

[44] Steering Committee for the Review of Government Service Provision (2013). Report on government services 2013.

[45] Mental Health Council of Australia, Recognition and respect (2012). Mental health carers report 2012.

[46] Lawn S, McNaughton D, Fuller L (2015). What carers of family members with mental illness say, think and do about their relative's smoking and the implications for health promotion and service delivery: a qualitative study. Int J Ment Health Promot.

[47] Mavundla TR, Toth F, Mphelane ML (2009). Caregiver experience in mental illness: a perspective from a rural community in South Africa. Int J Ment Health Nurs.

[48] El-Mallakh P, Yates BE, Adkins S (2013). Family caregiving for adults with schizophrenia and diabetes mellitus. Issues Ment Health Nurs.

[49] Bailey JM (2017). Family carers: a role in addressing chronic disease risk behaviours for people with a mental illness?. Prev Med Rep.

[50] Kitzinger J (1995). Qualitative research: introducing focus groups. BMJ.

[51] Morgan DL, Groups F (1996). Annu Rev Sociol.

[52] Schizophrenia Fellowship of NSW Inc. Carer assist: Support for mental health carers. 2008 [cited 2014 23.10.]; Available from: https://www.onedoor.org.au/services/carer-services.

[53] Guest G, Namey E, Mitchell M (2012). Collecting qualitative data: a field manual for applied research.

[54] QSR International. NVivo qualitative data analysis software. Version 11. Melbourne: QSR International Pty Ltd; 2012.

[55] Braun V, Clarke V (2006). Using thematic analysis in psychology. Qual Res Psychol.

[56] Borland R (2006). The potential of quitlines to increase smoking cessation. Drug Alcohol Rev.

[57] National Disability Insurance Agency (2013). What is the NDIS?.

[58] Happell B (2017). Physical health and mental illness: listening to the voice of carers. J Ment Health.

[59] Missen RL, Brannelly T, Newton-Howes G (2013). Qualitative exploration of family perspectives of smoke-free mental health and addiction services. Int J Ment Health Nurs.

[60] Royal College of Physicians and Royal College of Psychiatrists, Smoking and mental health (2013). Royal College of psychiatrists council report CR178.

[61] Australian Institute of Health and Welfare, National drug strategy household survey detailed report (2013). Drug statistics series no. 28. Cat. No. PHE 183.

[62] Diaz FJR, Velasquez DM, Susce DM, de Leon J MT (2006). smoking and smoking cessation among persons with severe mental illnesses. Psychiatr Serv.

[63] Thornton L (2011). Perceptions of anti-smoking public health campaigns among people with psychotic disorders. Mental Health Substance Use.

[64] Nonnemaker J (2014). The influence of antismoking television advertisements on cessation by race/ethnicity, socioeconomic status, and mental health status. PLoS One.

[65] Wye P (2010). Total smoking bans in psychiatric inpatient services: a survey of perceived benefits, barriers and support among staff. BMC Public Health.

[66] Connolly M (2013). Mental health nurses' beliefs about smoking by mental health facility inpatients. Int J Ment Health Nurs.

[67] Lancet Editorial (2013). Smoke alarm: mental illness and tobacco. Lancet.

[68] NSW Ministry of Health (2012). NSW tobacco strategy 2012–2017, N.M.o. Health.

[69] Government of South Australia, South Australia health (2013). Smoke-free policy, D.F.H.A. Ageing, editor.

[70] National Institute for Health and Care Excellence, Smoking cessation in secondary care (2013). Acute, maternity and mental health services.

[71] Bartlem K (2015). Acceptability and receipt of preventive Care for Chronic-Disease Health Risk Behaviors Reported by clients of community mental health services. Psychiatr Serv.

[72] Bartlem K (2014). Care provision to prevent chronic disease by community mental health clinicians. Am J Prev Med.

[73] Wye P (2010). An audit of the prevalence of recorded nicotine dependence treatment in an Australian psychiatric hospital. Aust N Z J Public Health.

[74] Wye P (2014). Observation of the extent of smoking in a mental health inpatient facility with a smoke-free policy. BMC Psychiatry.

[75] Prochaska JJ, Gill P, Hall SM (2004). Treatment of tobacco use in an inpatient psychiatric setting. Psychiatr Serv.

[76] McCann TV, Bamberg J, McCann F (2015). Family carers' experience of caring for an older parent with severe and persistent mental illness. Int J Ment Health Nurs.

[77] New South Wales Government (2014). NSW carers strategy 2014-2019.

[78] Happell B (2016). Physical health nurse consultant role to improve physical health in mental health services: a carer's perspective. Int J Ment Health Nurs.

[79] Parliament of Australia, National Disability Insurance Scheme, in 20, H. Families, Commnity Services and Indigenous Affairs, Editor. 2013, Parliament of Australia: Canberra.

[80] Independent Advisory Council (2014). IAC advice on implementing the NDIS for people with mental health issues, A.D.o.H. Services.

[81] COAG Disability Reform Council (2016). National Disability Insurance Scheme quartlerly actuarial report, A.D.o.H. Services.

[82] NSW Department of Health (2007). NSW carers action plan 2007–2012 summary, N.D.o. Health.

